# Wasting and Stunting in Infants and Young Children as Risk Factors for Subsequent Stunting or Mortality: Longitudinal Analysis of Data from Malawi, South Africa, and Pakistan

**DOI:** 10.1093/jn/nxab054

**Published:** 2021-04-08

**Authors:** Charlotte M Wright, John Macpherson, Ruth Bland, Per Ashorn, Shakila Zaman, Frederick K Ho

**Affiliations:** Department of Child Health, School of Medicine, Nursing and Dentistry, University of Glasgow, Glasgow, United Kingdom; Institute of Health and Wellbeing, University of Glasgow, Glasgow, United Kingdom; Institute of Health and Wellbeing, University of Glasgow, Glasgow, United Kingdom; School of Public Health, University of Witwatersrand, Witwatersrand, South Africa; Center for Child Health Research, Tampere University and Tampere University Hospital, Tampere, Finland; Department of Public Health, University of Health Sciences, Lahore, Pakistan; Institute of Health and Wellbeing, University of Glasgow, Glasgow, United Kingdom

**Keywords:** malnutrition, infant, young child, mortality, growth, stunting, wasting, outcomes, cohort study, lower- and middle-income countries

## Abstract

**Background:**

Few studies have had sufficient longitudinal data to track how different malnourished states relate to mortality at different ages and interrelate over time.

**Objectives:**

This study aims to describe the RRs and proportions of mortality associated with wasting and stunting and the pathways into and out of these nutritional states.

**Methods:**

Longitudinal growth data sets collected for children ages 0–24 months from Malawi, South Africa, and Pakistan were combined (*n* = 5088). Children were classified as deceased, wasted (weight for height < −2 SD; 1–4%), stunted (length < −2SD; 20–47%), or wasted and stunted (WaSt; 2–5%) at ages 3, 6, 9, 12, 18, and 24 months. Mixed-effects Cox models were used to study the association between nutritional status and mortality.

**Results:**

By age 3 months, 20% of children were already stunted, rising to 49% by 24 months, while wasting (4.2% and 2.2% at 3 months, respectively) and WaSt (0.9% and 3.7% at 24 months, respectively) were less common. The HR for mortality in WaSt was 9.5 (95% CI, 5.9–15), but 60% of WaSt-associated mortality occurred at 3–6 months. Wasting or WaSt was associated with 10–23% of deaths beyond 6 months, but in the second year over half of deaths occurred in stunted, nonwasted children. Stunting persisted in 82% of children and wasting persisted in 44%. Wasted children were more likely than nonwasted, nonstunted children to become stunted (RR, 1.93; 95% CI, 1.7–2.2), but 94% of children who progressed to stunting had not been wasted in the prior period.

**Conclusions:**

WaSt greatly increased the risk of death, particularly in very young infants, but more deaths overall were associated with stunting. Most stunting appeared to be either intrauterine in origin or arose in children without prior wasting. Either stunting and wasting represent alternative responses to restricted nutrition, or stunting also has other, nonnutritional causes.

## Introduction

Although much improved in many regions in recent years, child malnutrition remains a major issue in many parts of the world (1), which has implications for future adult health (2). In childhood, the term malnutrition implies either a chronic state of reduced growth (stunting) or the acute state of wasting, mainly due to a loss of (or failure to acquire) fat stores, but also of muscle mass (3). Most nutritional screening programs prioritize identifying wasting as a marker of acute undernutrition associated with the greatest risk of morbidity and death (4, 5). However, it has been suggested that stunting and wasting share common risk factors and that both should be considered together in program planning (3). Stunting that begins in early childhood is generally thought to be a later consequence of chronic undernutrition (6), and it is thus often assumed that wasting occurs early in the malnourished state and then progresses onto stunting. Previous studies have found suggestive associations between low weight or wasting and later stunting (7, 8), but others have noted that high rates of stunting may occur in settings where wasting is rare (9) and that stunting may have already occurred at birth (10). Most previous studies have lacked either the numbers or the detailed longitudinal data necessary to examine how children progress through different nutritional states over time. They were thus unable to determine whether each nutritional state poses the same risk at different ages. These studies described the RRs of different states, but for service planning purposes there is also a need to set these findings in the context of their respective prevalence, which may vary greatly by setting.

We have combined data from 3 large, longitudinal growth data sets with frequent measurements in the first 2 years from Malawi, South Africa, and Pakistan. This provides the size and diversity of data needed to allow us to consider a range of more detailed questions about the adverse consequences of different nutritional states, in terms of mortality and stunting. It is already known how important the different states are as risk factors for mortality, but how much of the total mortality is associated with different nutritional states, and to what extent are these age dependent? These detailed longitudinal data also allow us to consider the extent to which wasting or stunting persist over time, and what tends to precede and follow a period in each state. We can thus establish what the commonest consequences are of wasting and the commonest antecedents of stunting, and whether these transitions differ by age or setting.

## Methods

### Data sets

#### Lungwena Child Survival Study, Lungwena, Malawi

The Lungwena Child Survival Study was a cohort study of 795 women who were recruited in 1995–1996 and their newborn children, who were studied prospectively (11, 12). The cohort included mothers who were HIV positive (18%), and they were excluded from our analysis. Children were measured monthly to 18 months, then every 3 months up to 36 months. Children were measured at home by a research assistant using portable spring scales and length boards reading to 100 g and 0.5 cm. The scales and length boards were checked and calibrated regularly, and another assistant randomly repeated 5–10% of all measures. All deaths were recorded, but not their causes.

#### Africa Centre Vertical Transmission Study, South Africa

The Africa Centre Vertical Transmission Study study enrolled 2938 children, half of whom were born to HIV-infected women and half to uninfected women from 7 rural, 1 semi-urban, and 1 urban primary health care clinic in KwaZulu-Natal, into a nonrandomized intervention cohort study between 2001–2005 (13, 14). Our analysis included only the infants of the 1662 HIV-negative mothers. Measurements were collected by research staff, using the WHO-recommended protocol, monthly until 12 months, then every 3 months to 24 months. The mean of 2 weights was used unless there was a difference greater than 100 g, in which case a third measurement was taken and the 2 weights within 100 g were recorded. Length was measured to the nearest 0.1 cm. If the difference between the 2 measurements was greater than 5 mm, a third measurement was taken, and the 2 lengths within 5 mm were recorded. All deaths were recorded, with cause of death.

#### Lahore longitudinal study, Pakistan

The Lahore longitudinal study enrolled infants born between 1984–1994, with 1314 from a village area, 572 from a peri-urban slum, 921 from an urban slum, and 339 from a middle-class area, and followed them monthly from birth up to 36 months (15, 16). All infants were measured at home by specially trained research assistants, with the measuring technique checked monthly and instruments checked weekly (17). An acceptable precision was specified to be <100 g for weight and <1.0 cm for length and head circumference (15). All deaths were confirmed after a verbal autopsy conducted by the senior pediatricians.

#### Analysis

All 3 studies had a common basic sampling structure of monthly measurements collected for at least the first 12 months, measurements collected at least every 3 months thereafter, and all deaths recorded, with date of death. The 3 data sets could thus be combined. The measurements were compared to the WHO growth standard (18) to calculate weight-for-height (WFH) and length-for-age (LFA) z-scores. Children were then classified at each age point as wasted (WFH < −2 SD), stunted (LFA < −2 SD), wasted and stunted (WaSt = WFH < −2 SD and LFA < −2 SD), or dead.

The growth measurements and death data were both categorized as having occurred in 6 periods: from 2 weeks to <3 months, 3 to <6 months, 6 to <9 months, 12 to <18 months, and 18 to <24 months. If there were multiple measurements in a time period, the mean z-scores for all eligible measurements in that period were used. For analyses that did not require a defined period (multilevel Poisson regression and Cox regression), all the measurements were used.

As the Lahore data set was drawn from 4 distinct communities, the data set in total comprised 6 cohorts with varying socioeconomic and nutritional characteristics, and we set out at first to pool cohorts with similar growth patterns and mortality rates into fewer subsets, to give more statistical power for the between-group comparisons. It was felt reasonable to combine the Lahore peri-urban slum with the village data and the Lahore middle-class cohort with the South African cohort. This therefore meant that there were 4 subgroups: The least-deprived South Africa/Pakistan middle class cohort, the moderately-deprived Pakistani urban slum cohort, and the 2 very-deprived subgroups of the Malawi and the Pakistani Peri-urban/village cohorts (see **Supplemental Figure 1**).

Unadjusted RRs for mortality were calculated by age category. A mortality analysis was only conducted using the combined cohort data, because of insufficient events in individual cohorts. Mixed-effects Cox models were used to study the association between nutritional status and mortality rate. The outcome variable was time to event (either death or censoring). Nutritional status was treated as a time-varying predictor variable, adjusted for age at assessment and sex, and country-level correlation was modeled using random intercepts. We used 4 parametrizations of nutritional status: *1*) stunting only; *2*) wasting only; *3*) height-for-age and weight-for-height z-scores as continuous variables and their multiplicative interaction; and *4*) stunting only, wasting only, and stunting and wasting. HRs and 95% CIs were calculated to quantify the associations.

The associations between nutritional status in 2 consecutive periods were analyzed using multilevel Poisson regressions. The outcome variables were stunting and wasting, and the primary exposure variables of interest were stunting and wasting in the preceding period. Similar to the Cox models, these analyses were adjusted for age at assessment and for sex, and country-level correlation was modeled using random intercepts. Intrapersonal variation was additionally adjusted as random intercepts to account for underinflation, as the outcomes were binary (19). The transition probabilities between nutritional status in 2 consecutive periods were calculated, and CIs were derived assuming multinomial distributions. We regarded 2-sided *P* values < 0.05 as significant. All analyses were conducted using R 4.0.3 with packages “coxme” and “markovchain.”

## Results

There were 5088 individuals in total in the cohort, of whom 80% were alive and had growth data at 3 months, although by 24 months only 47% were alive and had growth data (Supplementary Figure 1). In total, 477 (9.4%) had died by 24 months; 170 (36%) of these deaths occurred shortly after birth and a further 28% occurred within the first 3 months.

The overall prevalence of stunting rose from 20% at 3 months to 49% by 24 months, while wasting fell from 4.2% at 3 months to 0.9% at 24 months. WaSt rose from 2.2% at 3 months to a peak of 5.2% at 12 months (**Table 1**).

**TABLE 1:** Nutritional status and mortality by age categories^1^

	Nutritional Status	Birth to 2 weeks	2 weeks to <3 mo	3 to <6 mo	6 to <9 mo	9 to <12 mo	12 to <18 mo	18 to 24 mo
Total combined	Wasted		4.2 (174)	3.5 (131)	2.9 (100)	2.3 (71)	1.5 (45)	0.9 (20)
	Stunted		20.4 (833)	22.6 (854)	28.0 (958)	33.6 (1049)	44.9 (1358)	49.1 (1184)
	WS		2.2 (90)	3.0 (115)	3.8 (132)	5.2 (162)	4.6 (138)	3.7 (90)
	Healthy		70.0 (2863)	69.1 (2609)	64.1 (2187)	58.3 (1823)	48.1 (1455)	45.6 (1100)
	Total	5088	100 (4090)	100 (3775)	100 (3417)	100 (3126)	100 (3027)	100 (2411)
Malawi	Wasted		0.2 (1)	0.4 (2)	0.5 (2)	1.0 (4)	0.5 (2)	0.3 (1)
	Stunted		49.7 (227)	62.9 (278)	68.3 (289)	73.1 (304)	77.5 (313)	74.4 (276)
	WS		0.2 (1)	0.4 (2)	1.2 (5)	2.9 (12)	2.5 (10)	3.5 (13)
	Healthy		46.4 (212)	33.3 (147)	27.4 (116)	21.1 (88)	16.8 (68)	19.1 (71)
	Total	523	100 (457)	100 (442)	100 (424)	100 (416)	100 (404)	100 (371)
Peri-urban/village	Wasted		8.4 (119)	6.7 (85)	5.7 (69)	4.0 (47)	1.7 (20)	0.5 (5)
	Stunted		18.8 (265)	22.3 (282)	31.4 (381)	38.8 (456)	56.1 (670)	61.7 (626)
	WS		4.7 (66)	7.0 (89)	8.8 (107)	10.9 (128)	9.6 (115)	6.2 (63)
	Heathy		62.6 (881)	61.3 (777)	52.8 (641)	45.4 (534)	32.5 (388)	31.1 (315)
	Total	1885	100 (1408)	100 (1268)	100 (1212)	100 (1175)	100 (1194)	100 (1014)
SA/middle class	Wasted		1.0 (14)	0.8 (11)	0.7 (7)	1.2 (10)	1.4 (11)	1.0 (5)
	Stunted		11.7 (162)	9.3 (120)	9.3 (97)	10.4 (88)	17.1 (132)	16.7 (82)
	WS		0.5 (7)	0.2 (3)	0.6 (6)	0.5 (4)	0.1 (1)	0.4 (2)
	Healthy		86.1 (1191)	88.9 (1147)	88.5 (927)	87.7 (742)	80.7 (623)	81.5 (402)
	Total	1759	100 (1383)	100 (1290)	100 (1047)	100 (846)	100 (772)	100 (493)
Urban slum	Wasted		4.7 (40)	4.3 (33)	3.0 (22)	1.4 (10)	1.8 (12)	1.7 (9)
	Stunted		21.2 (179)	22.4 (174)	26.6 (191)	29.3 (201)	37.6 (243)	37.5 (200)
	WS		1.9 (16)	2.7 (21)	1.9 (14)	2.6 (18)	1.8 (12)	1.8 (12)
	Healthy		68.8 (579)	69.4 (538)	68.5 (503)	66.6 (459)	58.1 (376)	58.5 (312)
	Total	921	100 (842)	100 (775)	100 (734)	100 (689)	100 (647)	100 (533)
Death	Total	3.4 (171)	3.2 (130)	1.7 (66)	1.2 (40)	0.7 (21)	1.0 (31)	0.7 (17)
	Malawi	5.9 (31)	3.5 (16)	2.9 (13)	2.6 (12)	1.9 (8)	2.7 (11)	2.7 (10)
	Peri/village	3.2 (61)	5.5 (77)	2.8 (34)	1.2 (14)	0.8 (10)	0.9 (11)	0.5 (5)
	SA/mid-class	3.7 (65)	0.6 (9)	0.7 (10)	0.9 (10)	0.2 (2)	0.6 (5)	0.4 (2)
	Urban slum	1.5 (14)	3.3 (28)	1.2 (9)	0.5 (4)	0.1 (1)	0.6 (4)	0 (0)

1 Numbers presented are percentages (*n*) unless otherwise specified. SA, South Africa; WS, wasted-stunted.

The 4 subgroups showed very different growth patterns over time. In the most prosperous group, wasting and WaSt were seen in only 1–2% at any age, but stunting rose from around 10% in the first year to 17% by 24 months. In the other 2 Pakistani cohorts, wasting alone was seen in 3–8% of children in the first 9 months, but in less than 2% in the second year. In the poorest Pakistani cohort, WaSt were seen in 5–11% of children and stunting rates rose from 19% at 3 months to 62% at 24 months. In contrast, in the Malawian cohort, stunting was extremely common at all ages, rising from 50% at 3 months to 78% at 18 months, but wasting alone was very rare (<1%).

### Likelihood of dying in relation to nutritional state

The fewest deaths occurred in the most prosperous subgroup and the most deaths occurred in the Malawi and peri-urban/village Pakistani subgroup, with the Malawi cohort experiencing more deaths from 12–24 months (Table 1). As nutritional status data were not generally available at birth, we were only able to examine how nutritional status related to deaths beyond the age of 3 months, as 133 (75%) children also had prior growth data (Table 1). Wasting and stunting were both associated with a much higher risk of death than the nonwasted, nonstunted (NWNS) state, with wasting showing roughly twice the risk of stunting and those with WaSt having the highest risk of all. The risk of WaSt was particularly high at the ages of 3–6 months (RR, 29.8; 95% CI, 14.0–63.4), albeit with wide CIs, and WaSt children made up nearly a quarter of all deaths in that period but much lower proportions at later ages (**Table 2**). Beyond 6 months, wasting or WaSt was associated with 10–23% of deaths, while 33–44% of deaths occurred in solely stunted children in the first year and 58–62% in the second year.

**TABLE 2:** Mortality by nutritional states in preceding period

Newly deceased at age, mo	% (*n*) of children in that state who died	Unadjusted RR (95% CI)^1^	% of all deaths at that age
Wasted-stunted			
3 to <6	13.5 (12)	29.8 (14–63)	24
6 to <9	2.6 (3)	4.0 (1.2–13.6)	10
9 to <12	0.8 (1)	2.8 (0.3–23.0)	6
12 to <18	1.2 (2)	3.3 (0.7–15.5)	8
18 to 24	1.4 (2)	10.6 (1.5–74.7)	15
Wasted			
3 to <6	2.4 (4)	5.2 (1.7–15.9)	8
6 to <9	0	0	0
9 to <12	2.0 (2)	7.5 (1.5–36.5)	13
12 to <18	1.5 (1)	3.9 (0.5–31.4)	4
18 to 24	2.4 (1)	17.9 (1.7–193)	8
Stunted			
3 to <6	2.5 (21)	5.6 (2.8–11.2)	42
6 to <9	1.2 (10)	1.8 (0.8–3.9)	33
9 to <12	0.7 (7)	2.7 (0.9–8.0)	44
12 to <18	1.4 (14)	3.5 (1.4–8.7)	58
18 to 24	0.6 (8)	4.3 (0.9–20.3)	62
NWNS			
3 to <6	0.5 (13)	Ref	26
6 to <9	0.7 (17)	Ref	57
9 to <12	0.3 (6)	Ref	38
12 to <18	0.4 (7)	Ref	29
18 to 24	0.1 (2)	Ref	15

1 Compared to NWNS (nonwasted nonstunted).

In a multivariable model adjusted for age and sex covariates, using intra-person and country variations as random intercepts, compared to NWNS children the overall HRs were 9.48 (95% CI, 5.94–15.13) for WaSt children, 4.35 (95% CI, 2.49–7.60) for wasted children, and 1.72 (95% CI, 1.16–2.55) for stunted children (**Table 3**). Each unit increase in the LFA or WFH z-score was associated with a 32% (95% CI, 22%–41%) or 45% (95% CI, 33%–54%), respectively, lower instantaneous risk of death, assuming a linear association. There was no multiplicative interaction between WFH and LFA z-scores. Restricting the reference group in the regression models to children with z-scores > −1 did not dramatically alter our findings, but this did increases the risk of stunting only and reduce that of wasting (**Supplemental Table 1**).

**TABLE 3:** Adjusted association of growth status and all-cause mortality in the next period^1^

	HR (95% CI)	*P*
Model 1		
Stunting	2.69 (1.93–3.76)	<0.0001
Model 2		
Wasting	5.42 (3.77–7.79)	<0.0001
Model 3		
LFA z-score	0.68 (0.59–0.78)	<0.0001
WFH z-score	0.55 (0.46–0.67)	<0.0001
Multiplicative interaction	0.96 (0.91–1.03)	0.27
Model 4		
Stunting only	1.72 (1.16–2.55)	0.007
Wasting only	4.35 (2.49, 7.60)	<0.0001
Stunting and wasting	9.48 (5.94, 15.13)	<0.0001

1 Adjusted for age and sex covariates, with intra-person and country variation as random intercepts in a mixed-effect Cox model. Model 1 used a binary variable comparing stunting (LFA < −2 SD) with nonstunting. Model 2 used a binary variable comparing wasting (WFH < −2 SD) with nonwasting. Model 3 had HFA and WFH z-scores as continuous variables and their multiplicative interaction terms. Model 4 used categorical variables with: nonstunting, nonwasting (reference group); stunting only; wasting only; and stunting and wasting. HFA, height for age; LFA, length for age; WFH, weight for height.

### Pathways from wasting

**FIGURE 1:**
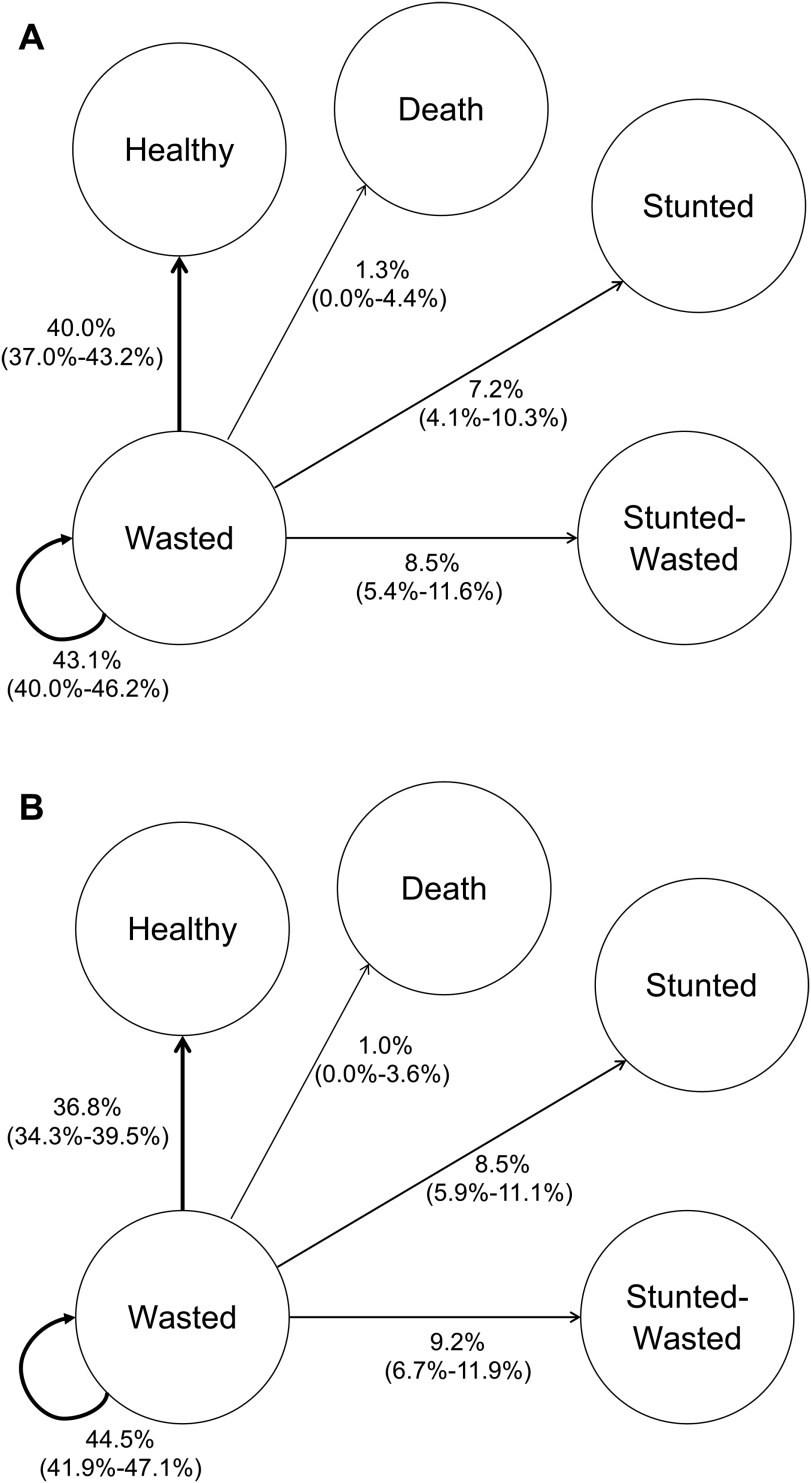
Pathways from wasting in a combined cohort (A: <1 year; B: ≥1 years). (A and B) Percentage values (95% CIs) of all wasted children moving to different states (NWNS, wasted, stunted, wasted-stunted) in the next 3-month period. The curved arrow indicates the percentage who remained in that state. NWNS, nonwasted nonstunted.

All children who were wasted in 1 period were, on average, most likely to move into a different state in the next period, most commonly moving back to being NWNS (**Figure 1**). These proportions differed little with age, but more so by subgroup (**Supplemental Figure 2**). In the most prosperous group, 56% of wasted reverted to being NWNS and only 9% progressed to being stunted, with or without wasting. In the other Pakistani subgroups, which had the highest prevalences of wasting, nearly half (50% urban slum and 45% peri-urban/village cohorts) of children remained wasted and 12–20% progressed to being stunted, with or without wasting. In contrast, in Malawi only 20% remained wasted, with more reverting to being NWNS, but also a third progressed to being stunted, with or without wasting.

### Pathways to and from stunting

**FIGURE 2:**
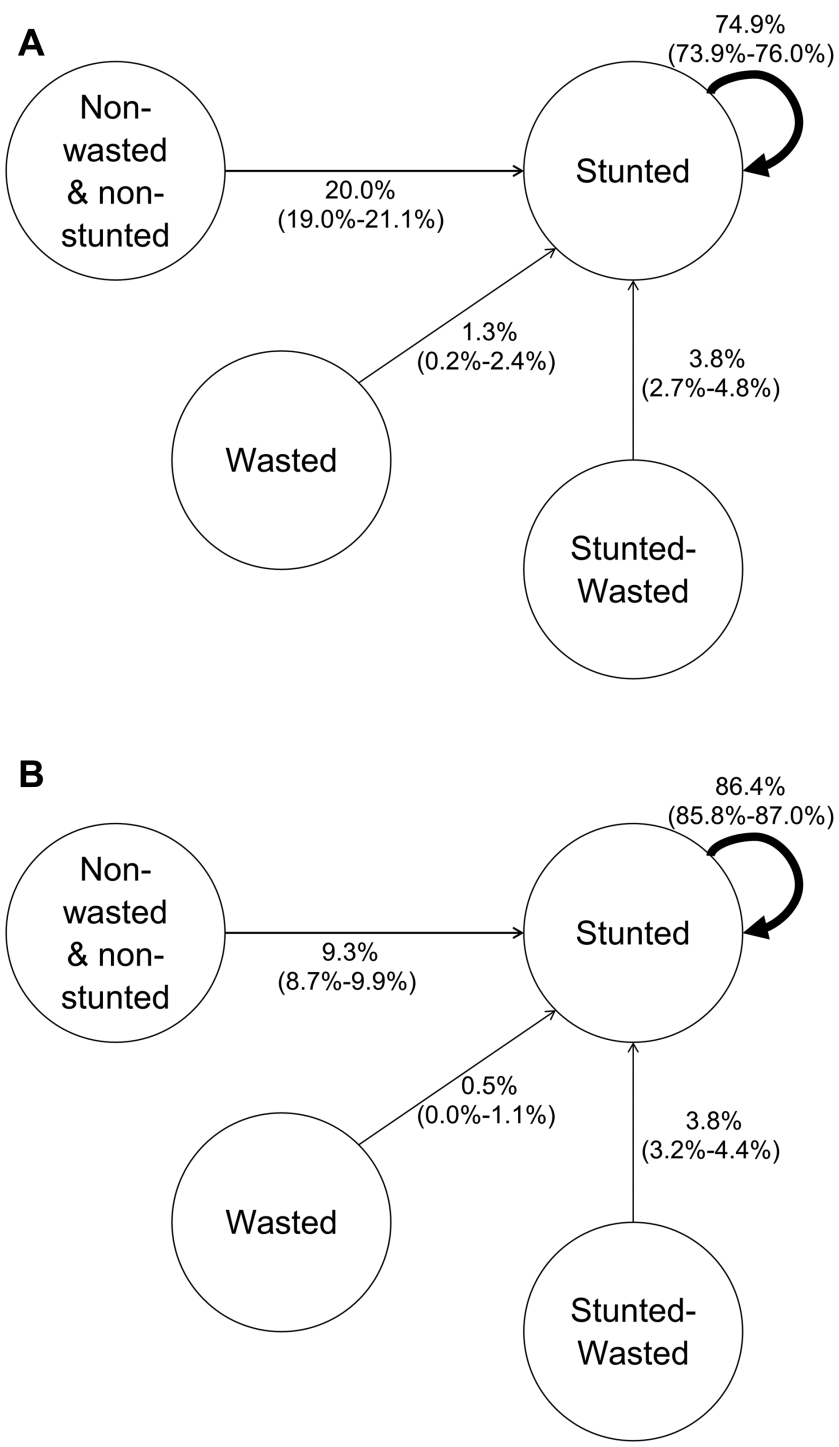
Pathways to stunting in combined cohort (A: <1 years; B: ≥1 years). (A and B) Percentage values (95% CIs) of all stunted children who had moved from each state in the preceding 3-month period. The curved arrow indicates the percentage who remained in that state.

Of those stunted (or WaSt) at age 18–24 months, 39% were already stunted at age 3 months. In the cohort with the most stunting (Malawi), 58% of those stunted at 24 months were already stunted by age 3 months. Overall, 75% of children under 1 year and 86% of children over 1 year remained stunted from a given 3-month period to the next (**Figure 2**). Most subgroups showed levels of persistence >80%, except for the most affluent subgroup, where in each period only 70% had persistent stunting (Supplemental Figure 2). Of those who were NWNS at 24 months, 22.5% had been stunted in at least 1 prior period.

Overall, wasted children were more likely (RR, 1.93; 95% CI, 1.69–2.20: *P* < 0.0001) than NWNS children to become stunted (**Supplemental Table 2**). However, because wasting was a much less common state, most children who progressed to stunting had been NWNS in the period before. This was the case for 97–98% of the most prosperous subgroup and the Malawi cohort, but was still true for 93% in the peri-urban/village cohort, which had the highest prevalence of wasting (**Supplemental Figure 3**). We repeated this analysis using mild wasting (WFH < −1SD) and having ever been wasted prior to becoming stunted, and >84% had still progressed to stunting from a nonwasted state (**Supplemental Figure 4**). If this was restricted only to children not already stunted by age 3 months, 75% had still never been wasted.

## Discussion

In this analysis, we aimed to describe how much total mortality is associated with wasting compared to stunting. As in previous studies, we were able to show that the risk of death after WaSt was very high, but that most of these deaths occurred before 6 months and a larger proportion of deaths at all ages were associated with stunting than with wasting. We also considered the extent to which wasting and/or stunting persist over time and what nutritional states tend to precede stunting. While wasted children were twice as likely as NWNS children to progress to stunting, most children progressing to stunting had not been wasted in the period before, even in settings where wasting was common.

This analysis combined data from a range of countries and socio-economic settings, whose heterogeneity ensured that we had substantial numbers in each state at most ages, even if they were supplied by different data sets at different ages. This also allowed us to explore the extent to which patterns of risk and recovery reflected the setting or the child's age.

There are many limitations to this analysis that must be acknowledged. It is likely to be more informative about the burden of stunting than that of wasting, as much of the data were collected from areas with high stunting rates but relatively lower rates of wasting, reflecting the inclusion of more affluent cohorts and of 1 African cohort with exceptionally low WaSt levels. The data were also all collected at a time when mortality and severe acute malnutrition were generally more common and when their management was perhaps less well organized. Nonetheless, the risks found here are comparable to those observed in a previous large meta-analysis (20). We do not know the specific causes of death for all cohorts, or detailed information on access to health care, but health care options would have been the same within each setting, so that the contrasts observed between nutritional states are still valid.

Wasting can be very acute, so it could have developed after the sampled measurement age and thus have been missed; if so, exposure to this state would have been relatively short. In this data set, wasting was generally most common in the first 3 months and became less common over the first year. This is in contrast to a study in 54 countries that found the lowest weight-for-length z-scores at 9 months (21). However, a recent study found a 3% prevalence of WaSt for children aged 6–59 months across 84 countries, similar to our rates overall (22).

This data set could not shed light on the correlates of growth immediately after birth, as there were not enough useable growth data at birth and gestation at birth was not reliably known. Thus, the impact of nutritional state on survival could only be considered for deaths occurring beyond 3 months, by which time around two-thirds of all deaths had already occurred. However, there were still enough deaths in the combined cohort to be informative. In 2 of the cohorts, HIV was prevalent and could have confounded any relationship between nutritional state and mortality, so data were excluded from children of all mothers known to be HIV positive. We do not know the HIV statuses of the Pakistani mothers, but at the time of the study the HIV prevalence was low in that region.

The purpose of this analysis was to compare the risks associated with stunting and wasting rather than their underlying causes, and we could not address underlying risk factors for either mortality or stunting. Although each cohort collected a range of socio-demographic and morbidity data, these were too diverse to be combined, as were data on infant feeding practices or dietary intakes.

The combination of wasting and stunting has already been recognized as more than additively increasing the risk of death (20, 23). What this study adds is the observation that most deaths following WaSt occurred in the first 6 months; thereafter, the risk was not much higher than for wasting alone. Wasting was clearly the more dangerous state relatively, but stunting, because of its much higher prevalence, was associated with twice as many deaths. This paradox was also noted in an earlier study (23).

It is well recognized that wasting is usually a transient state that, if survived, may be followed by a return to health (3). In contrast, stunting is generally assumed to be irreversible without a change of environment (24). However, recent studies have found substantial evidence of remission of stunting after 1 (25) or even 2 years of age (26, 27). In this cohort, most stunting did persist, but a minority of children reverted to being NWNS after each period; around 1 in 5 children who were not stunted at 24 months had previously been stunted.

A number of studies have previously presented evidence that wasting early in the malnourished state is associated with later stunting (7, 28, 29). In this data set, wasting was associated with a doubling of the risk of later stunting; nonetheless, most stunted children had never been wasted, and this remained true even in the subgroups with the highest prevalence of wasting. It has been noted that wasting and stunting rates are positively correlated in most countries, but in some countries there is no association (23) and 1 of the above studies also reflected that stunting was too common to be solely explained by wasting (7).

So what are the origins of stunting in the majority of children if stunting is not preceded by wasting? In our database, nearly 40% of children with sustained stunting were already stunted by the age of 3 months. Because we lack measurements at birth, we cannot explore in this analysis how much this relates to intra-uterine growth, as opposed to the period immediately after birth. Other studies have noted that a low birth weight and prematurity greatly increase the risk of subsequent stunting, as well as likely contributing to the high mortality in this period, so it seems likely that much of this reflects restricted intrauterine growth (30). While it might be hoped that these children would have recovered to a nonstunted state in ideal circumstances, in this cohort very few recovered. However, of the remaining children who were not stunted at 3 months but went to on become stunted, 75% had still never been wasted.

It is has been suggested that stunting and wasting might be alternative responses to undernutrition, rather than 1 being the consequence of the other (3). This could reflect different genetically coded local responses to energy or other nutrient depletion at the growth plate (31). However, if that was the main explanation, then the stunting prevalence should be substantially reversed by the provision of high-energy foods within preventive supplementary feeding programs or by treatment for moderately acute malnutrition. Sadly, while these programs generally impact on weight, they have had modest effects at best on height, even where great care has been taken to ensure good compliance (32).

This thus raises the question of whether other possible mechanisms need to be considered. The concept of environmental enteropathy, resulting from repeated exposure to and colonization of the gut by pathogenic bacteria, is also thought to be an important cause of stunting (33). While this could impact children via the mechanism of malabsorption and undernutrition, it is also possible that exposure to recurrent and repeated environmental pathogens could result in a chronic inflammatory state that itself may suppress growth, even in the absence of undernutrition, as has been shown in some disease states (34). For example, the Mal-Ed (Malnutrition and Enteric Disease)study found that enteropathogen exposure was an independent predictor of height growth, while actual episodes of diarrhea were not (35). So far, programmatic water, sanitation and hygeine interventions to prevent stunting have been just as disappointing as supplementary feeding (36), but these programs on the whole were not able to deliver the large-scale hygiene infrastructure changes that are required (37), and a recent review found that improvements in sanitation conditions were included among the large-scale changes associated with reductions in stunting (38).

## Conclusions

Both wasting and stunting were associated with increased risks of mortality, but the assumption that stunting is usually a result of chronic undernutrition is challenged by its limited association with wasting at an individual level. This further suggests that meeting the challenge of stunting requires a more multifaceted approach.

## Supplementary Material

nxab054_Supplemental_File

## Notes

This work was initially supported by Medical Research Council PhD studentship grant number MR/K500847/1 DTG. The Lungwena Child Survival Study was funded by the Academy of Finland, Emil Aaltonen Foundation, Foundation for Paediatric Research, Medical Research Fund of Tampere University Hospital, the Research Foundation of Mannerheim League for Child Welfare, and the Research Foundation of the University of Tampere. The Africa Centre Vertical Transmission Study was funded by Wellcome Trust UK (063009/Z/00/2). The Lahore longitudinal study was funded by the Swedish Agency for Research Cooperation with Developing Countries and King Edward Medical College, Lahore, Pakistan.

Author disclosures: CMW, JM, RB, PA, SZ, and FKH, no conflicts of interest.

Supplemental Figures 1–4 and Supplemental Tables 1 and 2 are available from the “Supplementary data” link in the online posting of the article and from the same link in the online table of contents at https://academic.oup.com/jn/.

Abbreviations used: LFA, length-for-age; NWNS, nonwasted, nonstunted; WFH, weight-for-height; WaSt, wasted-stunted.
