# Supplementary material for: Wasting and Stunting in Infants and Young Children as Risk Factors for Subsequent Stunting or Mortality: Longitudinal Analysis of Data from Malawi, South Africa, and Pakistan
Source: J Nutr. 2021 Apr 8;151(7):2022–8. doi: 10.1093/jn/nxab054 (PMC8245889; doi:10.1093/jn/nxab054)
Supplement: nxab054_Supplemental_File [file nxab054_supplemental_file.docx]

**Supplementary Table 1 Adjusted association of growth status (alternative definition) and all-cause mortality in the next period**

|  | HR (95% CI) | P |
| --- | --- | --- |
| Model 1 |  |  |
| Mild stunting | 1.89 (1.18-3.04) | 0.009 |
| Model 2 |  |  |
| Mild wasting | 1.53 (0.78-2.97) | 0.21 |
| Model 4 |  |  |
| Mild stunting only | 2.64 (1.53-4.58) | 0.0005 |
| Mild wasting only | 2.40 (1.10-5.21) | 0.027 |
| Mild stunting and mild wasting | 10.05 (5.72-17.67) | <0.0001 |

Adjusted for age and sex covariates, and intra-person and country variation as random intercepts in mixed effect Cox model

HR: hazard ratio

Model 1: A binary variable comparing mild stunting (HFA < -1SD) with non-stunting

Model 2: A binary variable comparing mild wasting (WFH < -1SD) with non-wasting

Model 4: A categorical variable with: non-mildly stunting non-mildly wasting (reference group), mildly stunting only, mildly wasting only, and mildly stunting and mildly wasting

**Supplementary Table 2 Adjusted association of nutritional status in two consecutive periods**

|  | **RR (95% CI)** | **P** |
| --- | --- | --- |
| Stunting predicted by: |  |  |
| Prior stunting in all children | 9.50 (9.10, 9.92) | <0.0001 |
| Prior wasting in nonstunted children | 1.93 (1.69, 2.20) | <0.0001 |
| Wasting predicted by: |  |  |
| Prior stunting in nonwasted children | 1.67 (1.49, 1.88) | <0.0001 |
| Prior wasting in all children | 13.68 (12.10, 15.45) | <0.0001 |

Adjusted for age and sex covariates, and intra-person and country variation as random intercepts in multilevel Poisson regression

RR: risk ratio

**Supplementary Figure 1: Participants flow diagram**


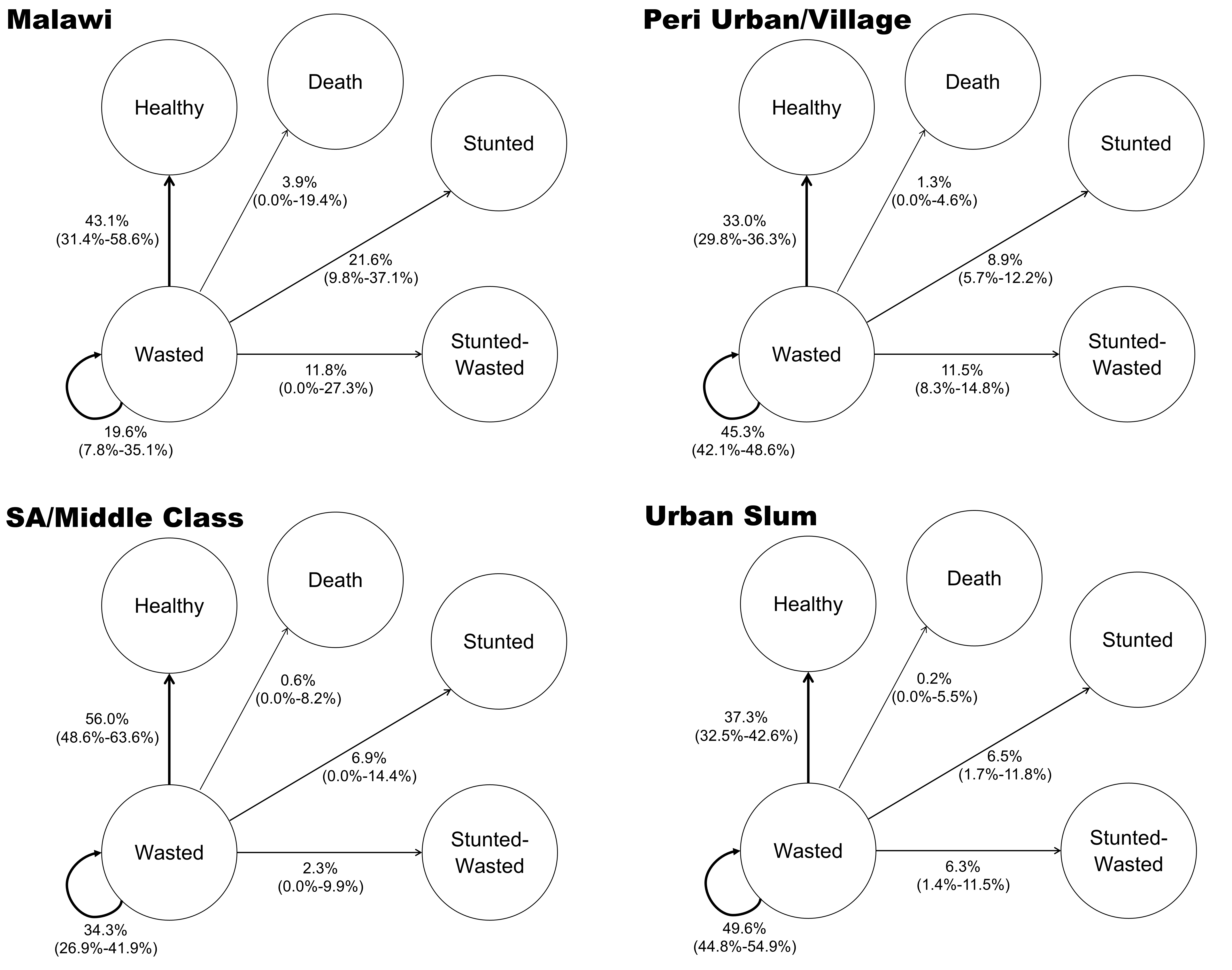


**Supplementary Figure 2: Pathways from wasting by sub-group**

Values are % (95% CI) of all initially wasted children moving to different states (non-wasted-non-stunted [NWNS], wasted, stunted, wasted-stunted) in the next 3 months period. The curved arrow indicted the % who remained in that state.


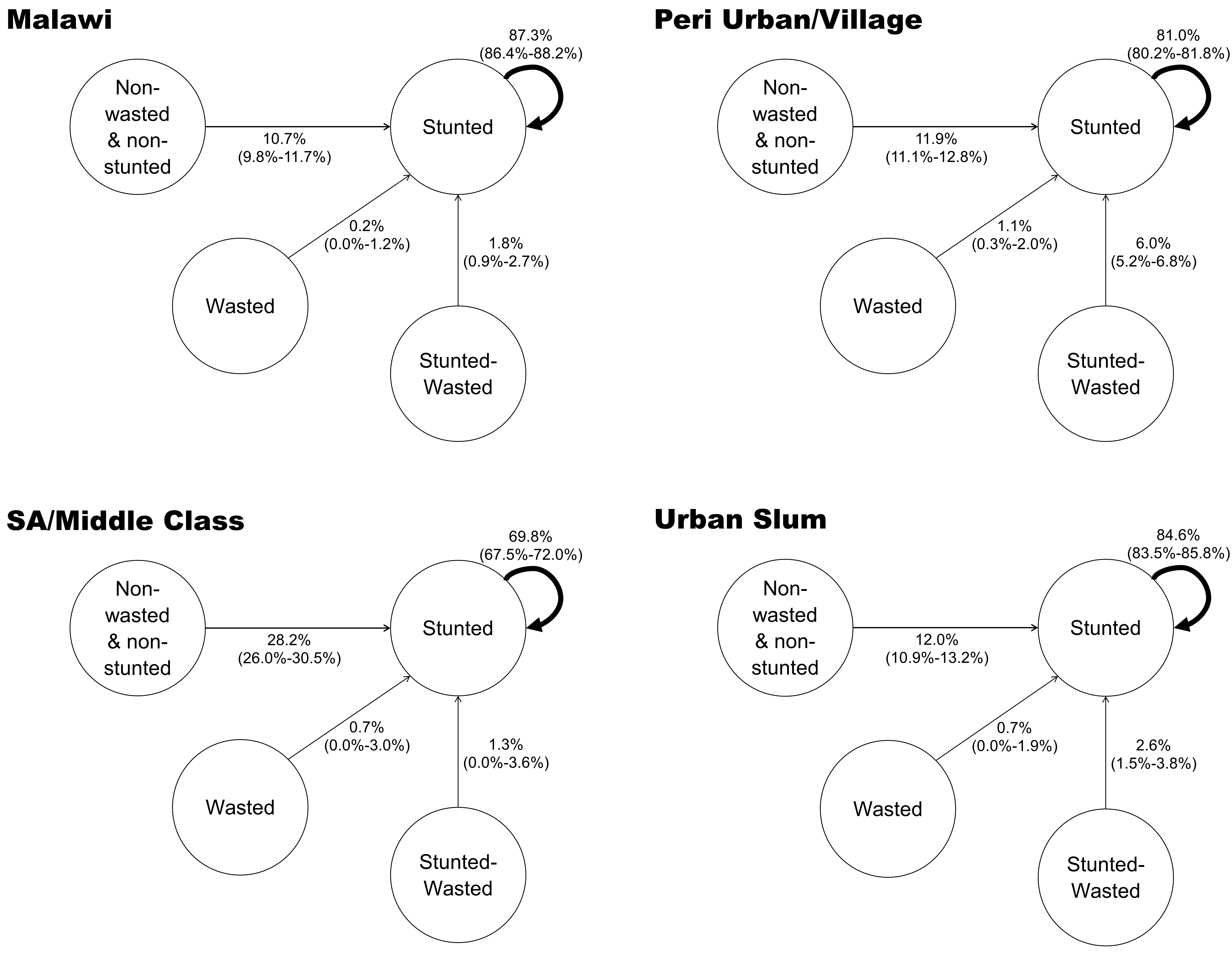


**Supplementary Figure 3: Pathways to stunting by sub-group**

Values are % (95% CI) of all stunted children who had moved from each state (non-wasted-non-stunted [NWNS], wasted, stunted, wasted-stunted) in the preceding 3 months period. The curved arrow indicates the % who were already stunted.


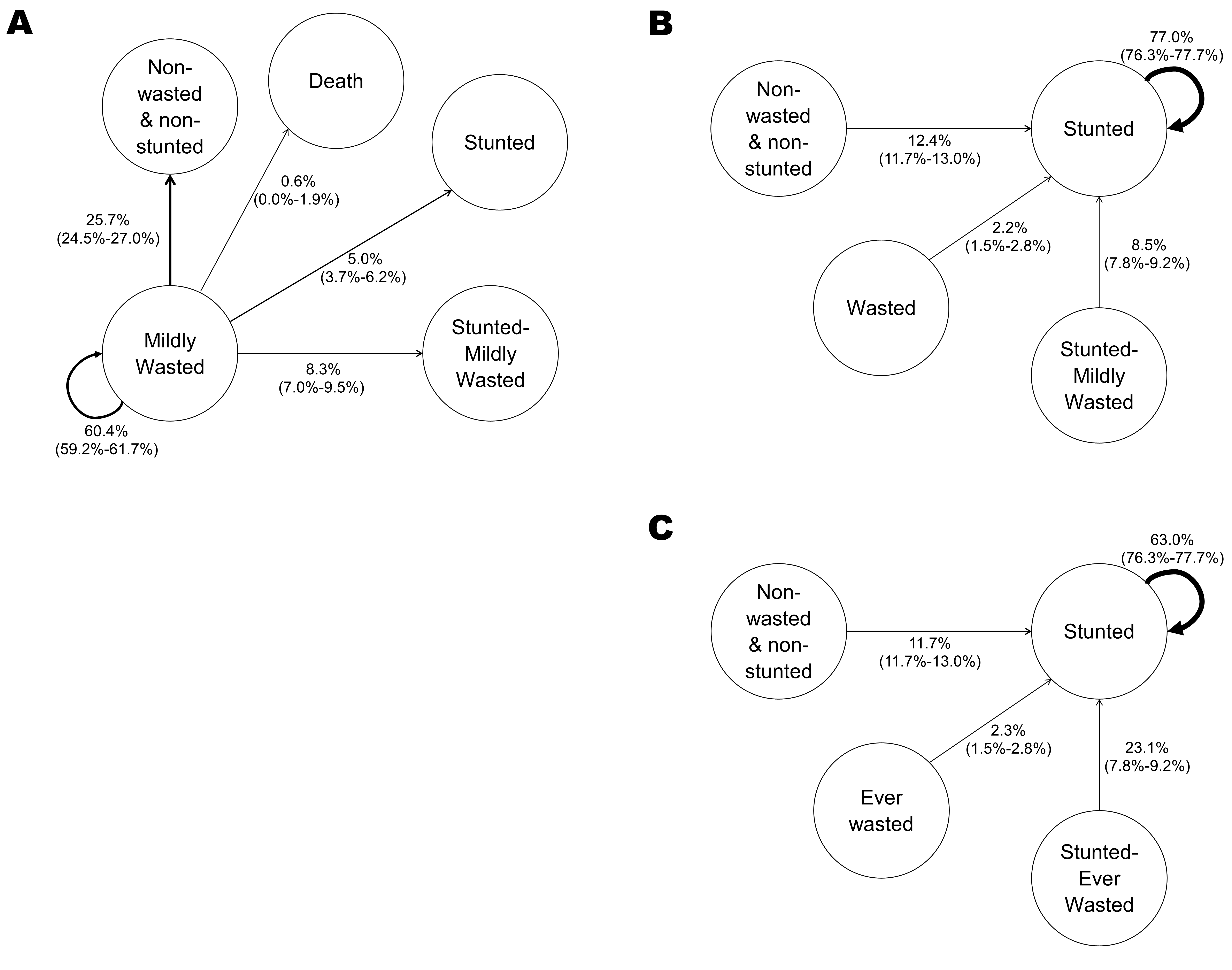


**Supplementary Figure 4: Sensitivity analyses for mildly wasted (A: from wasting; B: to stunting) and for ever wasted (C)**

Values are % (95% CI) of all stunted children who had moved from each state (non-wasted-non-stunted [NWNS], ever wasted, stunted, ever wasted-stunted) in the preceding 3 months period. Once a child was categorised as wasted, he/she was categorised as ‘ever wasted’ or ‘ever wasted-stunted’ depending on whether he/she was stunted. The curved arrow indicates the % who were already stunted.
